# Comparative Analysis of Quality of Life Determinants Among Various Cancer Patient Groups in Western Maharashtra, India

**DOI:** 10.7759/cureus.66353

**Published:** 2024-08-07

**Authors:** Rajani Patil, Girish Suragimath, Siddhartha Varma, Sameer A Zope, Ashwinirani SR

**Affiliations:** 1 Periodontology, School of Dental Sciences, Krishna Vishwa Vidyapeeth (Deemed to be University), Karad, IND; 2 Oral Medicine and Radiology, School of Dental Sciences, Krishna Vishwa Vidyapeeth (Deemed to be University), Karad, IND

**Keywords:** india, palliative care, western maharashtra, quality of life, general well-being, cancer patients

## Abstract

Introduction

Cancer is a major health problem and a devastating disease. People living with cancer experience a variety of signs and symptoms. Cancer patients undergo physical, psychological, social, and financial implications due to the disease and its treatment. Cancer harshly affects the individual’s health and deteriorates the quality of life (QoL). This study assesses the QoL determinants among various cancer patient groups in western Maharashtra, India.

Materials and methods

This hospital-based cross-sectional study was conducted in the oncology center, Krishna Vishwa Vidyapeeth, Karad, India. The study consisted of 270 cancer patients selected by purposive sampling technique. The data regarding the QoL was collected using a structured and validated questionnaire consisting of 50 questions elicited through one-to-one interviews. Data were analyzed using statistical software.

Results

Of 270 cancer patients, 135 (50%) were males and 135 (50%) were females aged between 22 and 66 years. Maximum patients (N = 89, 32.9%) were in the age group of 41-50 years, and the majority of patients (N = 34, 12.6%) suffered from breast cancer. The QoL among our patients was very low in 104 (38.5%) and 127 (47.0%), average in 36 (13.3%), and high only in 3 (1.1%) patients.

Conclusion

QoL among cancer patients was influenced by their symptoms, treatment, and the financial strain they experienced. There is a need to develop interventions to effectively manage symptoms that will help patients gain a greater sense of control over their illness and treatment, thereby improving their QoL.

## Introduction

Cancers are a group of diseases that cause abnormal cell growth, which invades other parts of the body and greatly affects every essence of quality living. Malignant cells can invade and destroy normal body tissue and spread to other parts of the body, which is known as metastasis. This invasion disrupts the affected tissues and organs and significantly impacts the overall quality of life (QoL) of individuals diagnosed with the disease. Cancer is also the leading cause of morbidity and mortality across the world.

The estimated number of incident cases of cancer in India for the year 2022 was found to be 14,61,427. One in nine people in India is likely to develop cancer in his/her lifetime [1]. Cancer diagnosis and treatment introduce plenty of challenges, significantly impacting all the domains of QoL. Cancer causes various signs and symptoms, which range from subtle to noticeable. The type, size, and stage of the cancer and its potential impact on vital organs or systems influence QoL [2].

The QoL refers to how much a person can enjoy the valued possibilities of their lives [3]. QoL is a critical aspect of healthcare, particularly for cancer patients, as it encompasses physical, emotional, and social well-being. Health-related QoL (HRQOL) encompasses the impact of health status on QoL [4]. The HRQOL of an individual deteriorates when he/she is diagnosed with cancer, as well as when they are undergoing treatment, which ultimately affects their overall QoL. The assessment of HRQOL in cancer patients will shed light on their sufferings and help healthcare professionals offer holistic support and enhance patient care. With this background, the present study aimed to assess the determinants of QoL among various cancer patient groups in Western Maharashtra, India.

## Materials and methods

Study design

This hospital-based cross-sectional study was conducted in the oncology center, involving 270 patients diagnosed with different types of cancers. The study employed the questionnaire adopted from the European Organisation for Research and Treatment of Cancer (EORTC QLQ-C30) to assess relevant parameters [5]. The patient information sheet was provided, and informed consent was obtained from all the patients. The study included subjects who were undergoing various cancer treatments for different types of cancer and had provided their consent to participate. Patients who did not provide informed consent and patients with communication barriers were excluded from the study.

Sample size estimation

The sample size for this study was calculated based on the results of the pilot study and on the level of significance (alpha error) of 5% and power of 80% using the statistical formula: N = (Z1)^2^[P(1 - P)]/d^2^, (N = 270).

Ethical issues

Ethical clearance was obtained from the institutional ethical committee of Krishna Vishwa Vidyapeeth (reference no: 202/2022-2023 dated: 17.1.2023). The study was conducted according to the Modified Helsinki Declaration [6].

Pilot study

A pilot study was conducted among 30 cancer patients using a questionnaire consisting of 50 questions to check the authenticity of the results. The results of the pilot study determined the reliability and validity of the pre-tested questionnaire. Of 30 cancer patients, six patients with breast cancer showed poor QoL, followed by esophageal, colorectal, and oral cancers. The pilot study results demonstrated that the questions employed were both valid and reliable. Additionally, the analysis revealed variability in responses across different groups of cancer patients, indicating that the type of cancer greatly influences the QoL. That is why we considered the type of cancer as an element in our questionnaire.

Collection of questionnaire data

A one-to-one interview with cancer patients or family members was employed to collect the data regarding the HRQOL. The assessment tool used in this study was the Marathi version of the European Organisation for Research and Treatment of Cancer (EORTC QLQ-C30) questionnaire [7].

Analysis of data

Statistical analysis was performed using IBM SPSS Statistics, version 22.0 (IBM Corp., Armonk, NY). Data analysis was performed using descriptive and inferential statistics. The obtained data were analyzed and compared statistically using an ANOVA test. The p-value of <0.05 was considered significant.

## Results

The mean age of the 270 patients was 45.56 ± 8.78 (mean ± standard deviation) years. The majority of patients were in the age group of 41-50 years (N = 89, 32.96%), followed by the age group of 51-60 years (N = 84, 31.11%), 31-40 years (N = 81, 30%), 21-30 years (N = 10, 3.7%), and above 60 years (N = 6, 2.22%). We considered an equal number of male (N = 135, 50%) and female (N = 135, 50%) patients in our study to reduce the gender bias of our results. The educational status of the patients showed that the majority of individuals were graduates and post-graduates (N = 173, 64.07%), followed by intermediate and post-high school (N = 61, 22.5%), high school (N = 18, 6.67%), primary school (N = 12, 4.44%), and middle school (N = 60, 2.22%). Distribution of patients according to socioeconomic status showed that the majority of patients were from the upper middle class (N = 84, 31.11%), followed by lower middle (N = 65, 24.07%), upper class (N = 62, 22.96%), upper lower class (N = 51, 18.89%), and lower class (N = 8, 2.96%).

Distribution of patients according to type of cancer showed that the majority of patients were suffering from breast cancer (N = 34, 12.59%), followed by stomach cancer (N = 32, 11.85%) (Table 1).

**Table 1:** Distribution of patients according to type of cancer

Cancer	Frequency	Percentage
Bladder	12	4.44
Brain	05	1.85
Blood	09	3.33
Breast	34	12.59
Colon	17	6.29
Colorectal	20	7.40
Endometrial	05	1.85
Kidney	10	3.70
Liver	24	8.89
Lung	19	7.03
Esophagus	07	2.59
Oral	18	6.67
Pancreatic	05	1.85
Prostate	10	3.70
Rectum	16	5.92
Stomach	32	11.85
Thyroid	05	1.85
Uterine	22	8.15
Total	270	100

The general well-being parameters of the patients were assessed, and we found that the majority of patients had moderate overall QoL (N = 228, 84.4%). All patients had moderate overall physical condition during the past month (Table 2).

**Table 2:** Distribution of patients according to general well-being parameters

Parameters	Very much, N (%)	Moderate, N (%)	Little, N (%)	Not at all, N (%)
How do you rate your overall quality of life during the past month?	00	228 (84.4%)	42 (15.6%)	00
How would you rate your overall physical condition during the past month?	00	270 (100%)	00	00
Do you feel you are physically performing less than what you want to do?	00	159 (58.9%)	111 (41.1%)	00
Do you feel confident about managing your financial needs in any situation?	00	142 (52.6%)	128 (47.4%)	00
Do you experience any pain at present?	00	111 (41.1%)	159 (58.9%)	00
Does your pain interfere with your day-to-day activity?	00	142 (52.6%)	128 (47.4%)	00
Is your appetite normal?	00	270 (100%)	00	00
Do you have any problem sleeping peacefully?	00	59 (21.6%)	181 (67.1%)	30 (11.1%)
Do you feel you need more rest?	00	87 (32.2%)	183 (67.8%)	00
Do you feel fatigued?	170 (62.9%)	100 (37.1%)	00	00
Are you able to move around (physically) as usual?	34 (12.6%)	117 (43.3%)	119 (44.1%)	00
Do you have problems in passing urine?	00	116 (42.9%)	154 (57.1%)	00
Do you have problems in passing motion?	00	128 (47.4%)	142 (52.6%)	00
Are you satisfied with your working capacity?	00	128 (47.4%)	142 (52.6%)	00

The psychological and cognitive well-being parameters among patients showed that the majority of patients felt moderate (N = 141, 52.2%) about themselves. Mostly (N = 155, 57.4%) patients had no difficulty remembering things. The majority of patients were dependent (N = 180, 66.7%) on medication (Table 3).

**Table 3:** Distribution of patients according to psychological and cognitive well-being parameters

Parameters	Very much, N (%)	Moderate, N (%)	Little, N (%)	Not at all, N (%)
How important do you feel about yourself at present?	41 (15.2%)	141 (52.2%)	88 (32.6%)	00
Have you had difficulty remembering things?	00	00	115 (42.6%)	155 (57.4%)
How dependent are you on medication?	180 (66.7%)	90 (33.3%)	00	00

The distribution of patients according to QoL showed that 47.04% (N = 127) had a low QoL, 38.52% (N = 104) had a very low QoL, and 13.33% (N = 36) had an average QoL (Table 4).

**Table 4:** Distribution of patients according to quality of life

Quality of life (score)	Frequency (N)	Percentage (%)
Very high (>82.5)	00	00
High (73.5-82.5)	03	01.11
Average (59-73)	36	13.33
Low (49.5-58.5)	127	47.04
Very low (<49.5)	104	38.52
Total	270	100

Table 5 shows the association between QoL and demographic variables. It was observed that age shows no statistically significant association with QoL among cancer patients (p > 0.05 by ANOVA test). Similarly, sex and socioeconomic status show no statistically significant association with QoL among cancer patients (p > 0.05 by ANOVA test).

**Table 5:** Association of quality of life and demographic variables

Demographic variables		QOL score (mean ± SD)	p-value
Age group (years)	21-30	106.12 ± 12.17	0.38 (NS)
	31-40	105.23 ± 11.21	
	41-50	104.19 ± 8.78	
	51-60	104.29 ± 10.14	
	>60	104.21 ± 10.10	
Sex	Male	105.29 ± 12.47	0.71 (NS)
	Female	105.71 ± 12.10	
Socioeconomic status	Upper class	102.14 ± 13.92	0.19 (NS)
	Upper middle class	103.13 ± 12.28	
	Lower middle class	104.13 ± 12.51	
	Upper lower class	104.33 ± 11.78	
	Lower class	106.19 ± 11.31	
(p-value >0.05: statistically not significant)			

Table 6 shows the association between QoL and type of cancer. We observed that type of cancer shows a statistically significant association with QoL among cancer patients (p < 0.05 by ANOVA test).

**Table 6:** Association of quality of life and type of cancer

Cancer	QOL score (mean ± SD)	p-value
Bladder	105.18 ± 10.11	0.03 (significant)
Brain	104.12 ± 10.18	
Blood	110.12 ± 10.23	
Breast	103.11 ± 10.18	
Colon	107.18 ± 12.24	
Colorectal	108.12 ± 14.19	
Endometrial	102.10 ± 10.21	
Kidney	105.23 ± 11.16	
Liver	107.18 ± 12.18	
Lung	108.45 ± 13.44	
Esophagus	107.29 ± 12.67	
Oral	104.12 ± 12.18	
Pancreatic	106.11 ± 12.12	
Prostate	101.19 ± 12.23	
Rectum	105.29 ± 10.13	
Stomach	106.45 ± 10.82	
Thyroid	105.21 ± 10.88	
Uterine	102.24 ± 7.36	
(p-value <0.05: statistically significant)		

## Discussion

Cancer is one of the major health issues and a devastating disease that affects all aspects of a healthy life. Various cancers impacting multiple organs are the leading causes of death worldwide. Cancer patients experience numerous symptoms throughout their illness, significantly affecting their QoL. In the present study on evaluating the QoL among different cancer types, it has been observed that both the specific type of cancer and the various treatment modalities exert differential impacts on patients’ QoL. Our hospital-based cross-sectional study assessed the QoL among 270 cancer patients at an Oncology center, comparing various types of cancers. Most patients were 41-50 years old (N = 89, 32.9%), consistent with Alam et al.’s findings [8]. Most of our participants (N = 173, 64.07%) completed higher secondary education, reiterating that educated people are more concerned about their health and seek early diagnosis and treatment for their medical problems [8].

In the EORTC QLQ-C30, higher scores on the Global Health Status/QoL scale correspond to better QoL. This is similar to the interpretation of our study, where higher scores correspond to better QoL [5]. Our study found that almost all patients (N = 267, 98.8%) had below-average QoL scores, heavily influenced by their symptoms. Patients reported very low general well-being (N = 228, 84.4%), had low self-esteem (N = 141, 52.2%), faced memory recall difficulties (N = 115, 42.6%), and relied heavily on medication for symptom relief (N = 180, 66.7%). These results align with Nayak et al.’s study conducted in India [9]. The findings of our study were also consistent with Gandhi et al., who conducted a cohort study involving 100 patients with advanced incurable head and neck cancer [10]. The study revealed that the patients experienced various symptoms, such as pain, insomnia, loss of appetite, and fatigue, which significantly impacted their normal functioning. Emotional functioning was affected in about 52.2% (N = 141) of the patients, while almost 80% (N = 216) of the population experienced a significant impact on their physical functioning [10].

Pain affected 52.6% (N = 141) of participants’ physical well-being, fatigue impacted 62.9% (N = 170), sleep problems were reported by 67.1% (N = 181), and appetite issues were almost universal. Notably, 59.8% (N = 159) felt they performed moderately well physically, and 52.6% (N = 142) were moderately confident in managing their financial needs. Pain, sleep problems, and fatigue were the most common issues affecting daily activities. Most participants had moderate problems with urination (N = 154, 57.1%) and bowel movements (N = 142, 52.6%). Despite numerous advancements in cancer treatment, the prevalence of physical and emotional symptoms remains high and can significantly impact the QoL of cancer patients [11]. Lin et al. assessed the pattern of symptoms in patients with advanced head and neck cancer in Taiwan and found that the most common symptom experienced was weight loss, followed by pain, cough, dysphagia, feeding difficulties, and communication difficulties [12]. Analysis done by Siemens et al. showed that one-fifth of the newly diagnosed advanced cancer patients experienced moderate or severe anxiety and depression symptoms and a very low QoL [13]. In our study, we observed that a significant proportion of patients experienced low levels of psychological well-being, which was similar to the study conducted by Marzo et al. [14].

In the current study, financial constraints are reported as the major issue among patients as well as family caregivers, which is the most common barrier to symptom management and has a bigger impact on QoL. Similar findings were reported in the studies by Alawadi and Ohaeri, Hopwood et al., Härtl et al., and Singh et al. [15-18]. Anxiety/depression and other symptoms were found to affect all dimensions of QoL, as reported by Bužgová et al. [19]. Cancer patients (N = 228, 84.4%) rated their overall QoL as moderate during the previous week and moderately poor physical condition during that week. Kannan et al. found that 80% of their study population had average or below-average QoL, similar to our findings [20]. Breast cancer patients have been observed to have low QoL scores in this study. Similarly, Knobf et al. found that breast cancer patients experienced low QoL scores due to changes in their body image [21]. Enien et al. conducted a study on Egyptian women with breast cancer and reported lower overall QoL in breast cancer survivors [22]. Our study identified cancer type as a significant factor affecting QoL, reporting moderate overall QoL. These results are in agreement with the study conducted by Deb Barma et al., who reported moderate QoL in head and neck cancer patients with significant functional impairments [23]. Similarly, Kumar et al. also observed moderate QoL, with cancer type being a major factor affecting QoL, whereas age, gender, and socioeconomic status have less influence on QoL. Psychological well-being and medication dependency are consistent across both studies [24]. Bhattacharjee and Ghosh emphasized spirituality and education as key predictors, with rural patients experiencing better QoL but facing severe social QoL challenges [25].

Limitations and future perspective

Limitations of our study include a small sample size and focus on just one region, plus relying on people’s own reports, which can be biased. The study only looks at a single point in time, missing long-term trends and other influencing factors. Future studies should involve a larger and more varied group of people, use various types of data, and monitor changes over time. Taking into account additional factors and cultural differences will provide a better understanding of how cancer impacts QoL.

## Conclusions

This study highlights that cancer patients suffer from various symptoms affecting their QoL. Managing cancer pain is crucial, and health professionals need to be sympathetic toward these patients and help them overcome the physical, psychological, and financial constraints they experience. Healthcare professionals must develop effective measures for symptom management and empower patients with strategies for better control over their illness and treatment, improving their QoL.
